# The Prognostic Role of mTOR and P-mTOR for Survival in Non-Small Cell Lung Cancer: A Systematic Review and Meta-Analysis

**DOI:** 10.1371/journal.pone.0116771

**Published:** 2015-02-13

**Authors:** Lei Li, Dan Liu, Zhi-Xin Qiu, Shuang Zhao, Li Zhang, Wei-Min Li

**Affiliations:** 1 Department of Respiratory Medicine, West China Hospital, Sichuan University, Chengdu, P.R. China; 2 Lab of Pathology, Department of Pathology, West China Hospital, Sichuan University, Chengdu, P.R. China; University de Minho, PORTUGAL

## Abstract

**Objectives:**

The mammalian target of rapamycin (mTOR) and phosphorylated mTOR (p-mTOR) are potential prognostic markers and therapeutic targets for non-small cell lung cancer (NSCLC). However, the association between mTOR/p-mTOR expression and NSCLC patients’ prognosis remains controversial. Thus, a meta-analysis of existing studies evaluating the prognostic role of mTOR/p-mTOR expression for NSCLC was conducted.

**Materials and Methods:**

A systemically literature search was performed via Pubmed, Embase, Medline as well as CNKI (China National Knowledge Infrastructure). Studies were included that reported the hazard ratio (HR) and 95%CI for the association between mTOR/p-mTOR expression and NSCLC patients’ survival. Random-effects model was used to pool HRs.

**Results:**

Ten eligible studies were included in this meta-analysis, with 4 about m-TOR and 7 about p-mTOR. For mTOR, the pooled HR of overall survival (OS) was 1.00 (95%CI 0.5 to 1.99) by univariate analysis and 1.22 (95%CI 0.53 to 2.82) by multivariate analysis. For p-mTOR, the pooled HR was 1.39 (95%CI 0.97 to 1.98) by univariate analysis and 1.42 (95%CI 0.56 to 3.60) by multivariate analysis.

**Conclusion:**

The results indicated that no statistically significant association was found between mTOR/p-mTOR expression and NSCLC patients’ prognosis.

## Introduction

Lung cancer is the leading cause of cancer-related death all over the world during the past few years. Non-small cell lung cancer (NSCLC) represents the most frequent type, with a percentage of 80–85% of all primary lung carcinomas[1]. With the continuous researches on the mechanism of carcinogenesis, treatments such as chemotherapy, radiotherapy and surgery have improved a lot. However, the 5-year survival rate is still not exceeding 15% until now[2]. Current knowledge regards NSCLC as the outcome of changes in several signaling pathways. Therefore, managements of some key therapeutic targets may help to predict and improve the prognosis of NSCLC[3].

Several therapeutic targets have drawn public attention, such as EGFR[4], HER2[5] and KRAS[6]. However, problems on drug resistance and gene mutation rate limit their development. In recent years, another potential candidate, phosphatidylinositol 3-kinase/v-akt murine thymoma viral oncogene homolog 1/mammalian target of rapamycin pathway (PI3K/Akt/mTOR pathway) emerges. It plays a critical role in cell survival, growth, proliferation, motility, as well as metabolism [7–9]. The mammalian target of rapamycin (mTOR) consists of two independent functional complexes: mTORC1 and mTORC2. They are phosphorylated by Akt1 at Ser2448. Then phosphorylated mTOR (p-mTOR) will activate P70S6K and inhibit 4EBP1, regulating ribosome biogenesis and protein synthesis[10]. Dysregulation of mTOR signaling pathway, which is due to genetic variation of several key genes, has bene observed in different types of cancers, such as urothelial bladder cancer[11], breast cancer[12], hepatocellular carcinoma[13] and lung cancer[14].

During the past few years, effects of dysregulation of mTOR pathway has been intensively investigated for NSCLC. However, the assocication between prognosis and mTOR/p-mTOR expression is still controversial. According to the contradictory results gathering from different studies, this meta-analysis was conducted to assess the prognostic value of mTOR/p-mTOR expression for NSCLC patients.

## Methods

### 1. Literature search strategy

Pubmed, Embase, Medline (ovid), Cochrane Library as well as CNKI (China National Knowledge Infrastructure) was searched comprehensively for relevant articles published until July 5, 2014. The search terms were used as follows: (1). mTOR or mammalian targets of rapamycin; (2). lung tumor or lung cancer or lung carcinoma or lung neoplasm; (3). survival or prognosis or prognostic.

### 2. Eligibility criteria

All languages were included, but articles with abstract only were excluded because of insufficient information. Titles and abstracts were examined at first to eliminate not applicable studies, such as studies on animals or cell lines, reviews and studies about other diseases. Then all remaining articles were screened carefully for eligibility. Articles were included in this meta-analysis if they met the following criteria: (1). Proven diagnosis of NSCLC; (2). Immunohistochemistry (IHC) was used to measure the expression level of mTOR and p-mTOR; (3). The correlation of mTOR/p-mTOR expression and patients’ overall survival (OS) were analyzed; (4). Hazard Ratio (HR) and 95% confidence interval (CI) were provided or could be calculated. (5). If the same cohort of patients were analyzed in more than one studies, only the most recent and complete study could be included. Either abstracts or full texts were examined by two reviewer (Lei Li and Dan Liu) independently. Disagreements were resolved by discussion with the third reviewer (Li Zhang).

### 3. Data extraction

Data were extracted from eligible studies by two reviewers (Lei Li and Dan Liu) independently with a predefined table. The following variables were retrieved: first author, publication year, country where the study was conducted, sample size, histological type, HR with its 95%CI (univariate and multivariate analysis). Quality of the eligible studies was assessed with the European Lung Cancer Working Party quality scale for biological prognostic factors for lung cancer[15].

### 4. Statistical analysis

HR was used as the effective index to describe the impact of mTOR/p-mTOR expression on overall survival of the patients. Positive mTOR/p-mTOR expression inticated poor survival if HR>1 and its 95%CI did not overlap with 1. Some studies presented HR and 95%CI directly. In other studies, Kaplan-Meier survival curves were used to calculate these values, with a software named Engauge Digitizer Version 4.1 (free software from http://digitizer.sourceforge.net/). This method was reported by Parmar MK[16] and has been widely used in meta-analysis for survival endpoints[17,18]. Then individual HRs were extracted to calculate pooled HR. A fixed-effects model or random-effects model was used according to the heterogeneity analysis.

Q test and I^2^ test were used to measure heterogeneity among studies[19], while funnel plot and Begg’s test were used to estimate the potential publication bias[20]. Moreover, sensitivity analysis and subgroup analysis were also conducted. All p values in this meta-analysis were two tailed, and P<0.05 was considered statistically significant. STATA 12.0 (Stata Corporation, College Station, Texas) was used to conduct the statistical analysis.

## Result

### 1. Literature search

**Figure 1:**
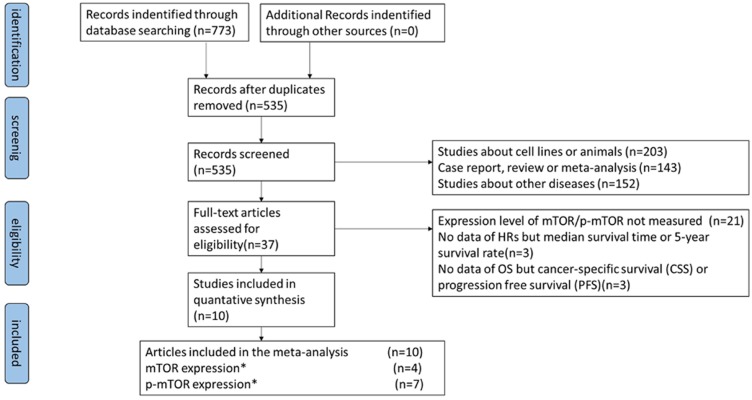
The literature searching process. (*) One article reported both the mTOR and the p-mTOR expression level.

A flowchart of our literature searching process is shown in Fig. 1. Using the searching strategy above, 773 entries were retrieved. After removal of 238 duplicate articles, 535 titles and abstracts were screened carefully. Thirty-seven articles appeared to be eligible for this meta-analysis. Of the remaining articles, 27 studies were ruled out because of the following reason: expression level of mTOR/p-mTOR not measured (21), no data about HRs but median survival time or 5-year survival rate (3), no data about OS but cancer specific survival (CSS) or progression free survival (PFS). Eventually, 10 articles were eligible for this meta-analysis, including 4 about mTOR expression and 7 about p-mTOR expression.

**Study characteristics**. Characteristics of 4 studies on mTOR were shown in Table 1. These articles were published between 2009 and 2012. A total of 614 participants were involved. Two of these studies were launched in Europe, 1 in USA and 1 in Asian. All articles reported HRs and 95%CI directly. Except for 1 study, other researches reported both univariate analysis and multivariate analysis.

**Table 1:** Characteristics of studies on mTOR expression.

First authe	Country			year	No. of patients (mTOR high/low)	Age (y)	Histological type(SCC/ADC/Other)	Stage (I/II/III/IV)	Univariate HR estimated	Univariate HR(95%CI)	Multivariate HR estimated	Multivariate HR(95%CI)
Tony D.[21]	Italy			2010	134(73/61)	> = 65y, 57 <65y, 77	56/41/37	91/43/0/0	HR 95%CI	1.77(1.17–2.73)	HR 95%CI	1.66(1.01–2.74)
Gately K.[22]	UK			2012	141(101/40)	> = 65y, 67 <65y, 74	67/60/14	97/44/0/0	HR 95%CI	1.85(0.98–3.49)	HR 95%CI	2.18(1.12–4.23)
Liu D.[23]	China			2011	172(106/28)	> = 60y, 91 <60y, 81	75/77/20	I-II,85 III-IV, 87	HR 95%CI	0.645(0.377–1.103)	NA	NA
Valsamo K.[24]	USA			2009	167(94/73)	64	NA	NA	HR 95%CI	0.44(0.22–0.88)	HR 95%CI	0.48(0.24–0.98)

Abbreviation: SCC, squamous cell carcinoma; ADC, adenocarcinoma; HR hazard ratio; NA, no available

Characteristics of 7 studies on p-mTOR were listed in Table 2. These studies were published between 2008 and 2014. Five studies were conducted in Asian and 2 in USA. A total of 1525 patients were enrolled. Two articles did not report HRs and 95%CI directly, so K-M curves were used to calculate these results.

**Table 2:** Characteristics of studies on p-mTOR expression.

First auther	Country	year	No. of patients (p-mTOR high/low)	Age (y)	Histological type(SCC/ADC/Other)	Stage (I/II/III/IV)	Univariate HR estimated	Univariate HR(95%CI)	Multivariate HR estimated	Multivariate HR(95%CI)
Yong Z.[25]	China	2013	120(58/62)	> = 60y, 66 <60y, 54	63/57/0	38/34/40/8	K-M curve	2.66(1.66–4.27)	HR 95%CI	2.642(1.157–4.904)
Hong-bing L.[26]	China	2008	59(24/35)	> = 60y, 28 <60y, 31	27/28/4	16/25/18/0	K-M curve	1.78(0.8–3.95)	HR 95%CI	0.686(0.274–1.721)
Akihiko Y.[27]	USA	2010	276(129/147)	NA	138/138/0	115/29/30/7	HR 95%CI	1.01 (0.75–1.36)	NA	NA
Liu D.[23]	China	2011	172(89/82)	> = 60y, 91 <60y, 81	75/77/20	I-II,85 III-IV, 87	HR 95%CI	1.917(1.349–2.724)	HR 95%CI	3.299(1.928–5.645)
Haruhisa K.[28]	Japan	2014	220(163/57)	65±9.0	78/148/4	126/43/49/2	K-M curve	0.55(0.28–1.27)	NA	NA
Kathryn A.[29]	USA	2014	370(NA)	65.7(32–90	126/227/17	234/75/61/0	NA	NA	HR 95%CI	0.662(0.460–0.952)
Shimizu K.*[30]	Japan	2014	104(41/63)	> = 65y, 73 <65y, 31	66/38/0	I-II,80 III-IV, 24	HR 95%CI	1.079(0.597–1.948)	NA	NA
Shimizu K.*[30]	Japan	2014	204(56/148)	> = 65y, 137 <65y, 67	142/62/0	I-II,159 III-IV, 41	HR 95%CI	1.475(0.868–2.505)	NA	NA

*Two independent groups were studied in this article, and HRs were reported separately.

Information about IHC criteria used to detect mTOR/p-mTOR expression of each study was listed in Table 3.

**Table 3:** IHC criteria used to detect mTOR/p-mTOR.

First author	Cut off value	antibody	company	dilution	Blinded reading
mTOR					
Tony D.[21]	>30 scores (IHS)	Rabbit monoclonal antibody	CST, Danvers, MA	1:50	Yes
Gately K.[22]	>30 scores (IHS)	Rabbit monoclonal antibody	CST, Danvers, MA	1:50	Yes
Liu D.[23]	> = 2 scores	Rabbit monoclonal antibody	CST, Beverly, MA	1:50	Yes
Valsamo K.[24]	>28 scores(IHS)	Rabbit monoclonal antibody	CST	1:50	NA
p-mTOR					
Yong Z.[25]	> = 2 scores	NA	CST	1:50	Yes
Hong-bing L.[26]	> = 2 scores	Ser2448, Rabbit monoclonal antibody	CST	1:100	NA
Akihiko Y.[27]	TS3, TS4, TS5	Ser2448	CST	1:100	Yes
Liu D.[23]	> = 2 scores	Ser 2448, Rabbit monoclonal antibody	CST, Beverly, MA	1:100	Yes
Haruhisa K.[28]	NA	NA	CST, Beverly, MA	1:100	NA
Kathryn A.[29]	> = 2 scores	NA	CST	1:100	Yes
Shimizu K.*[30]	> = 2 scores	Rabbit monoclonal antibody	CST, Danvers, MA	1:80	Yes

IHS: a semiquantitative immunohistochemical score used to assess both the intensity of staining and the percentage of positive cells; Blinded reading: readers of the slides without knowing clinical information.

### 2. Quality assessment

The quality of 10 included articles was assessed according to Lung Cancer Working Party quality scale for biological prognostic factors for lung cancer. Results were listed in Table 4. For the scientific design part, all of the articles represented study objectives, outcome definition and statistical methods distinctly, while preliminary assessment of the sample size was reported in none of these studies. For the other parts, scores differed obviously from each other. Seven articles didn’t test the reproducibility of the results, six didn’t present the tissue sample conservation.

**Table 4:** Quality assessment for the studies included in the meta-analysis.

First author	mTOR/p-mTOR	Scientific design(/10)	Laboratory methodology(/10)	Generalizability(/10)	Results analysis(/10)	Overall (%)
Tony D. [21]	mTOR	7.00	5.71	9.17	8.75	76.58
Gately K. [22]	mTOR	7.00	5.71	5.83	10.00	71.37
Valsamo K. [24]	mTOR	7.00	8.57	8.33	10.00	84.76
Yong Z. [25]	p-mTOR	7.00	5.71	7.50	8.75	72.41
Hong-bing L. [26]	p-mTOR	7.00	5.71	6.67	5.00	60.95
Akihiko Y. [27]	p-mTOR	7.00	6.43	6.67	10.00	75.24
Haruhisa K. [28]	p-mTOR	6.00	4.29	6.67	6.25	58.01
Kathryn A. [29]	p-mTOR	7.00	10.00	7.50	10.00	86.25
Shimizu K. [30]	p-mTOR	7.00	7.86	8.33	10.00	82.98
Dan L. [23]	mTOR&p-mTOR	7.00	8.57	7.50	10.00	82.68

### 3. Relationship of mTOR expression with survival

Of the four articles about mTOR expression, all were pooled into meta-analysis by univariate analysis, and 3 were included in meta-analysis by multivariate analysis because one study didn’t provide enough information.

**Figure 2:**
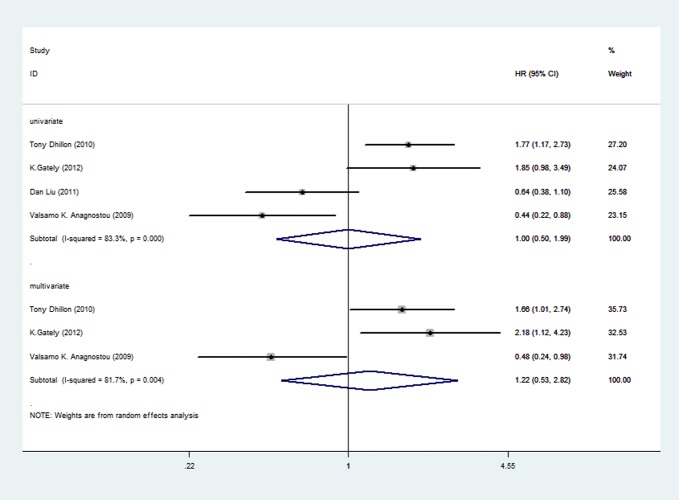
Forest plot showing the pooled HR of mTOR from random-effects model for overall survival by univariate and multivariate analysis

Four articles, including 614 participants, represented the HRs using univariate analysis. HRs for each study and pooled HR were shown in Fig. 2. The prognostic roles of mTOR/p-mTOR for NSCLC were inconsistent in different studies, with both negative and positive reported. The pooled HR was 1.00 (95%CI 0.5 to 1.99), indicating no statistically significant relationship between mTOR expression and overall survival. Random-effects model was used because of significant heterogeneity (P = 0.000, I^2^ = 83.3%) among researches. Three articles were included in the meta-analysis by multivariate analysis, with a total of 442 patients. The association between mTOR expression and prognosis remained no statistical significance, with the pooled HR of 1.22 (95%CI 0.53 to 2.82). Random-effects model was used because of significant heterogeneity (P = 0.004, I^2^ = 81.7%) among researches.

### 4. Relationship of p-mTOR expression with survival

**Figure 3:**
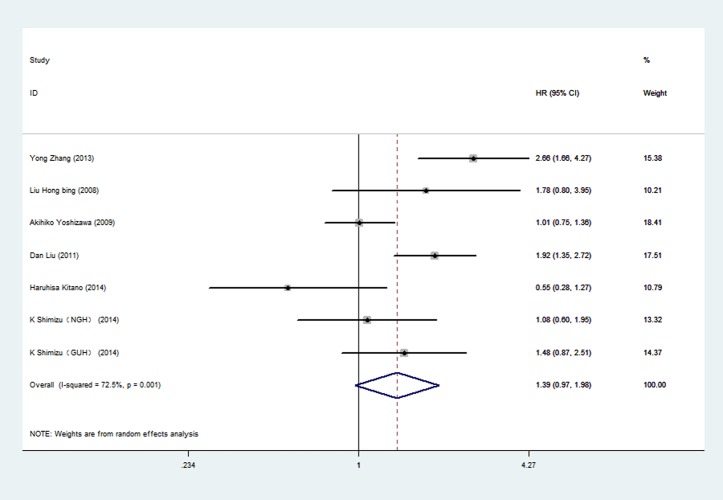
Forest plot showing the pooled HR of p-mTOR from random-effects model for overall survival by univariate analysis.

**Figure 4:**
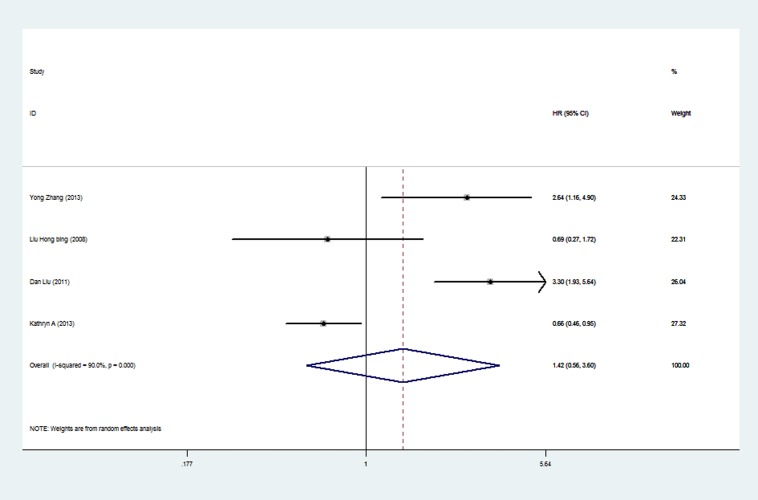
Forest plot showing the pooled HR of p-mTOR from random-effects model for overall survival by multivariate analysis.

Among the 7 articles about p-mTOR, six reported HRs by univariate analysis and 4 reported HRs by multivariate analysis. Meta-analysis was conducted by univariate analysis at first, including 1155 participants (Fig. 3.). The pooled HR was 1.39 (95%CI 0.97 to 1.98), indicating no statistically significant relationship between p-mTOR expression and overall survival. Random-effects model was used because of significant heterogeneity (P = 0.001, I^2^ = 72.5%) among researches. The pooled HR of p-mTOR by multivariate analysis was shown in Fig. 4. Four articles and 721 patients were included. The pooled HR was 1.42 (95%CI 0.56 to 3.60), indicating no statistically significant relationship between p-mTOR expression and overall survival. Random-effects model was used because of significant heterogeneity (P = 0.000, I^2^ = 90.0%) among researches.

### 5. Subgroup analysis

Because of too few articles about mTOR expression, stratifying analysis was only conducted for p-mTOR by univariate analysis. Main results of subgroup analysis were listed in Table 5 (Forest plots of each group were presented in S1, S2, S3, S4, S5 Figs.). Stratifying by geographic region, the pooled HR for studies conducted in China was 2.11 (95%CI 1.66 to 4.27) with less heterogeneity (p = 0.501, I^2^ = 0.0%), indicating that p-mTOR expression was significantly associated with poor prognosis; However, the condition was different in non-China subgroup (HR 1.03, 95%CI 0.83 to 1.30) with less heterogeneity (p = 0.217, I^2^ = 32.6%). Stratified analysis according to stage was also conducted; p-mTOR expression was associated with poor prognosis (HR 2.11, 95%CI 1.66 to 4.27) in studies with more I-II patients, with less heterogeneity (p = 0.501, I^2^ = 0.0%). The association between p-mTOR expression and poor prognosis remained statistically significant in subgroup with less ADC patients (HR 1.77, 95%CI 1.33 to 2.35). However, no statistically significant association between p-mTOR and prognosis was found in subgroup with number of patients more than 200 (HR 1.03, 95%CI 0.80 to 1.31).

**Table 5:** Subgroup analysis for p-mTOR by univariate analysis.

Factor	No. of study	No. of patients	HR	95%CI	Heterogeneity (P)	Heterogeneity (I^2^, %)
Geographic region						
China	3	351	2.11	1.66–4.27	0.501	0.0
Non-China	4	804	1.03	0.83–1.30	0.217	32.6
Stage I-II (%)						
> = 70	3	351	2.11	1.66–4.27	0.501	0.0
>70	4	804	1.03	0.83–1.30	0.217	32.6
Histological type (ADC %)						
> = 50	2	496	0.83	0.47–1.45	0.143	53.5
<50	5	659	1.77	1.33–2.35	0.187	35.2
HR estimated						
HR 95%CI	4	756	1.33	0.94–1.88	0.045	62.7
K-M curve	3	399	1.42	0.55–3.64	0.002	83.4
No. of patients						
<200	4	455	1.88	1.48–2.40	0.139	45.4
> = 200	3	700	1.03	0.80–1.31	0.109	54.8

When grouped by the way HR was provided, 4 articles listed HR and 95%CI directly, while 3 showed the K-M curves only. In both of the two subgroups, no statistically significant relationship was found between p-mTOR expression and prognosis, with significant heterogeneity.

### 6. Publication bias

**Figure 5:**
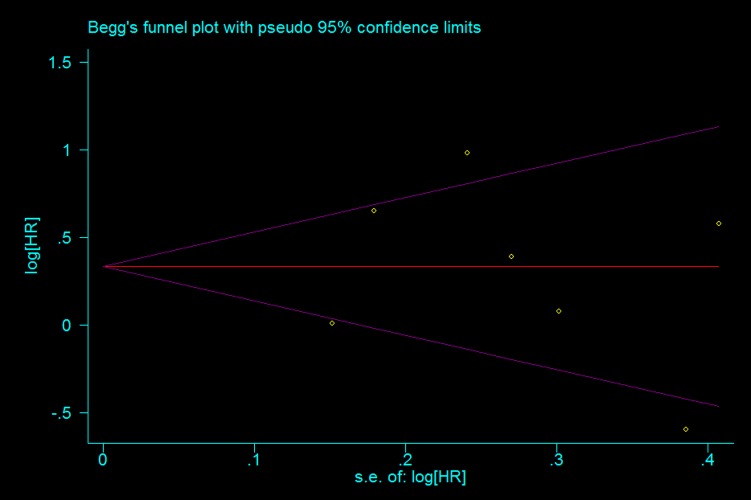
Begg funnel plot for publication bias test of the studies about p-mTOR expression by univariate analysis.

Begg’s test was performed to evaluate the publication bias (Fig. 5). No evidence of publication bias for p-mTOR by univariate analysis was found (p = 0.881).

### 7. Sensitivity analysis

**Figure 6:**
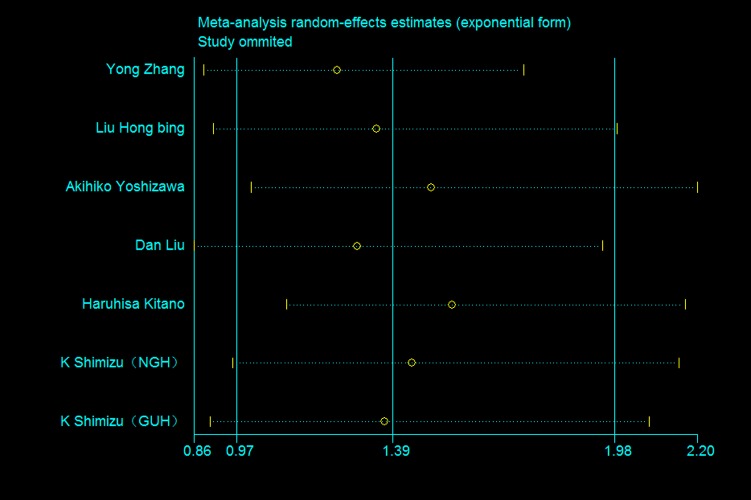
Sensitivity analysis of all the studies about p-mTOR expression by univariate analysis

Because of limiting number of articles using multivariate analysis, sensitivity analysis were performed in articles using univariate analysis. Results were shown in Fig. 6. One article was omitted at a time. The deletion of any individual article made no significant difference, indicating the robustness of the results in this meta-analysis.

## Discussion

The current meta-analysis summarized results of 10 articles, including 4 on mTOR expression with a total of 614 participants, and 7 on p-mTOR with 1525 cases. The pooled HR of overall survival indicated that no statistically significance was founded between mTOR/p-mTOR expression and NSCLC patients’ prognosis, using both univariate analysis and multivariate analysis, with significant heterogeneity. However, when stratified by geographical region, stage, histological type and number of patients, heterogeneity reduced significantly. Moreover, significant association was found between p-mTOR expression and poor prognosis in subgroups with more I-II patients, less ADC patients and studies conducted in China.

The prognostic role of mTOR/p-mTOR expression has been studied extensively in other types of cancers, despite results were still controversial. Xiaoyan Zhou et al[31]reported that p-mTOR expression increased when proliferation and invasion increased in breast cancer. Moreover, patients with high p-mTOR level had significantly shorter DFS. In gastric cancer, p-mTOR expression was closely linked to poor prognosis[32]. However, in Li Xiao’s research, cumulated survival rate of patients with mTOR expression was obviously higher than patients without its expression [33]. Thus, no consistent conclusion has been drawn about the prognostic significance of mTOR/p-mTOR in other cancers.

Moreover, mTOR inhibitors, such as deforolimus, everolimus and temsirolimus, has been accessed extensively in clinical trials for NSCLC. Thanyanan Reungwetwattana[34] reported that in a phase Ⅱ clinical trial with 52 frontline NSCLC patients, temsirolimus achieved a clinical benefit of 35% (8% confirmed PR and 27% with stable disease) as a single agent. In another clinical trial, everolimus monotherapy achieved 5.3% PR and a median PFS of 11.3 weeks in 85 patients with refractory advanced NSCLC[35]. These studies seemed inconsistent with results achieved in our meta-analysis. This paradox may result from the limited number of original articles included in this meta-analysis.

The current meta-analysis had several advantages. First, no publication bias was detected, and sensitivity analysis showed no significant difference when omitting any single article. Second, both mTOR and p-mTOR expression were studied, and both univariate and multivariate analysis were used in this meta-analysis.

Meanwhile, several limitations existed in this meta-analysis should also be presented. First, the number of studies involved was relatively small, especially studies about mTOR expression. Pooled HRs would be biased by this limitation either towards exaggeration or underestimation; furthermore, stratifying analysis were difficult to conduct because of insufficient information. Second, some HRs were extracted from K-M curves. Inaccuracy may generate when reading survival rates. Third, confounding factors inherent in these included articles may also bias the pooled HR markedly. Although most of the original studies adjusted for several known risk factors of NSCLC, many uncertain confounding factors could not be omitted. Thus, a more precise analysis with sufficient information was needed to adjust for covariates such as age, gender, smoking status, histological type and TNM stage. Forth, despite all of the studies included use IHC to detect mTOR/p-mTOR expression, cut-off points for positive and negative expression varied apparently. It might also impact the results. Finally, although publication bias were not found in this meta-analysis, the potential bias was still possible because articles with positive results tend to be published easier.

In summary, no statistically significant relationship was found between mTOR/p-mTOR expression and NSCLC patients’ prognosis. However, more high quality studies were needed to perform a more precise meta-analysis, exploring the prognostic significance of mTOR/p-mTOR expression in NSCLC.

## Supporting Information

S1 PRISMA Checklist PRISMA 2009 Checklist (DOC)

S1 PRISMA Diagram PRISMA 2009 Flow Diagram (DOC)

S1 Fig Forest plot of subgroup analysis stratifying by geographic region. (TIF)

S2 Fig Forest plot of subgroup analysis stratifying by stage. (TIF)

S3 Fig Forest plot of subgroup analysis stratifying by histological type. (TIF)

S4 Fig Forest plot of subgroup analysis stratifying by HR estimated. (TIF)

S5 Fig Forest plot of subgroup analysis stratifying by number of patients. (TIF)
