# Supplementary material for: The Prognostic Role of mTOR and P-mTOR for Survival in Non-Small Cell Lung Cancer: A Systematic Review and Meta-Analysis
Source: PLoS One. 2015 Feb 13;10(2):e0116771. doi: 10.1371/journal.pone.0116771 (PMC4332670; doi:10.1371/journal.pone.0116771)
Supplement: S1 PRISMA Diagram — (DOC) [file pone.0116771.s002.doc]

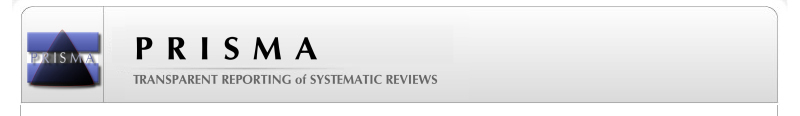
**PRISMA 2009 Flow Diagram**

**Screening**

**Included**

**Eligibility**

**Identification**

Records identified through database searching
(n = 773 )

Additional records identified through other sources
(n = 0 )

Records after duplicates removed
(n = 535 )

Records screened
(n = 535 )

Records excluded
(n = 493 )

Full-text articles assessed for eligibility
(n = 37 )

Full-text articles excluded, with reasons
(n = 27 )

Studies included in qualitative synthesis
(n = 10 )

Studies included in quantitative synthesis (meta-analysis)
(n = 10 )
