# Supplementary figures and images for: The Prognostic Role of mTOR and P-mTOR for Survival in Non-Small Cell Lung Cancer: A Systematic Review and Meta-Analysis
Source: PLoS One. 2015 Feb 13;10(2):e0116771. doi: 10.1371/journal.pone.0116771 (PMC4332670; doi:10.1371/journal.pone.0116771)

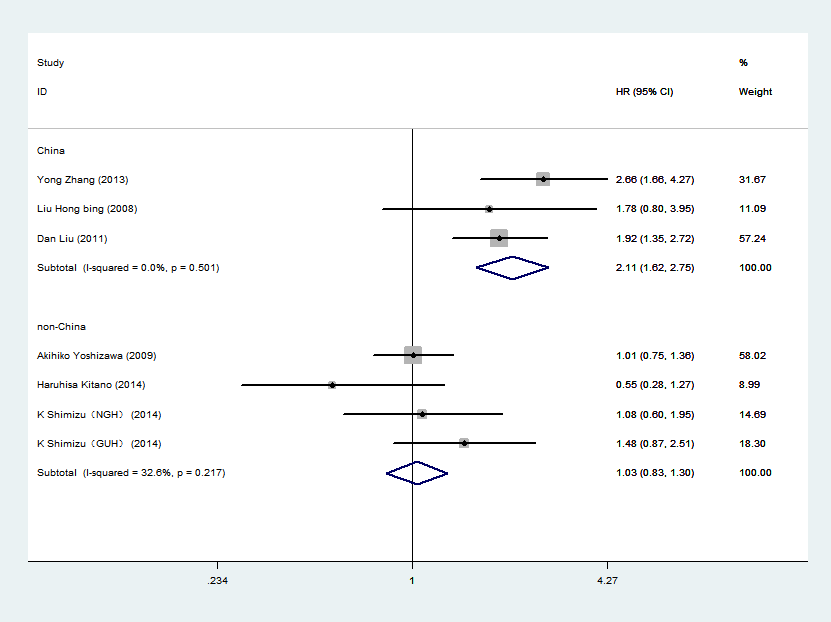

Supplement: S1 Fig — (TIF) [file pone.0116771.s003.tif]

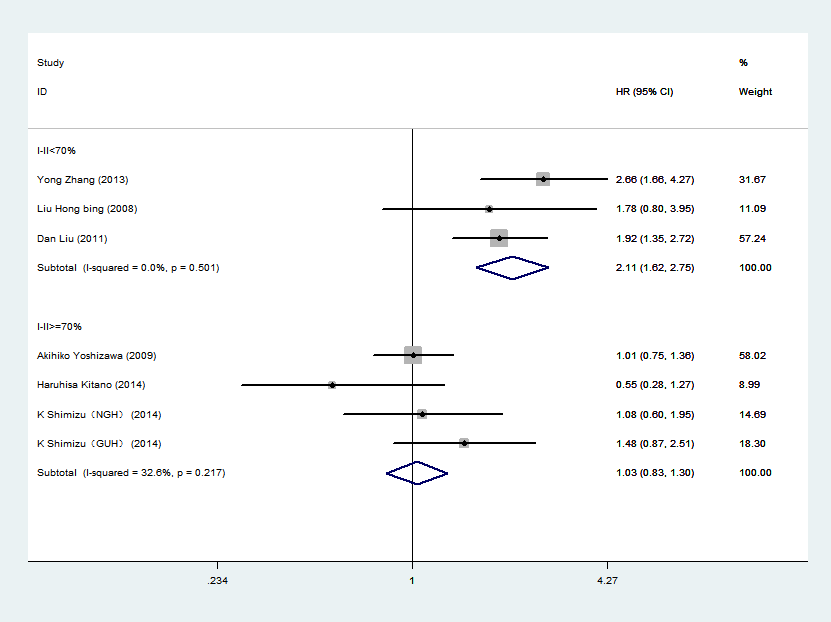

Supplement: S2 Fig — (TIF) [file pone.0116771.s004.tif]

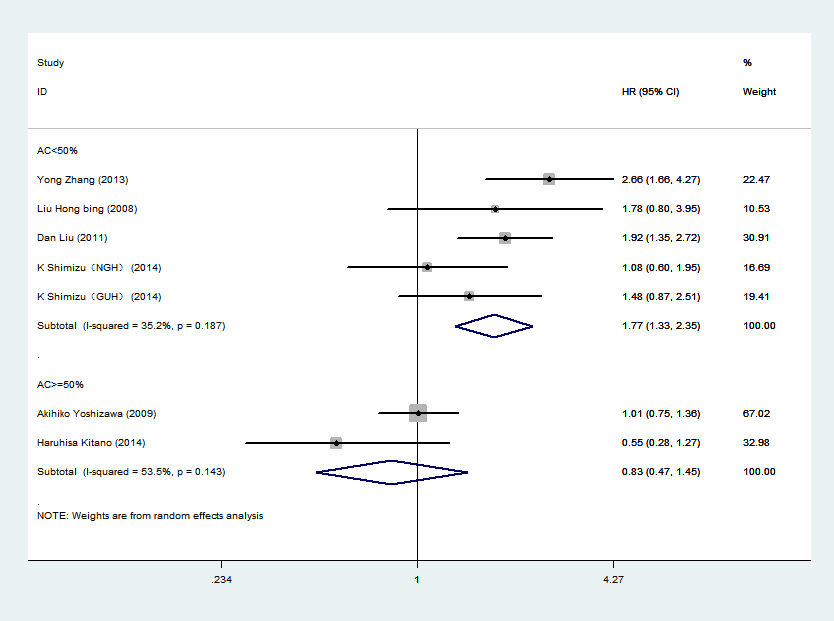

Supplement: S3 Fig — (TIF) [file pone.0116771.s005.tif]

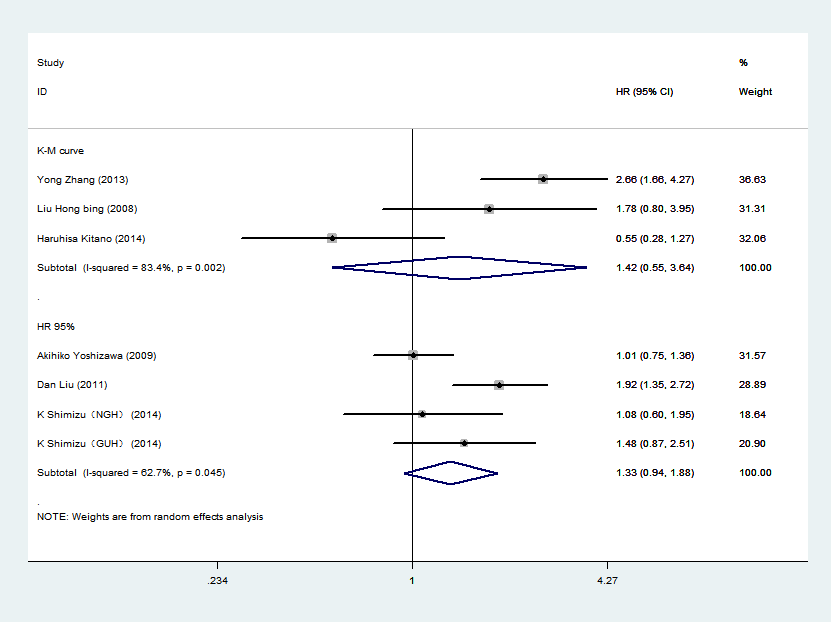

Supplement: S4 Fig — (TIF) [file pone.0116771.s006.tif]

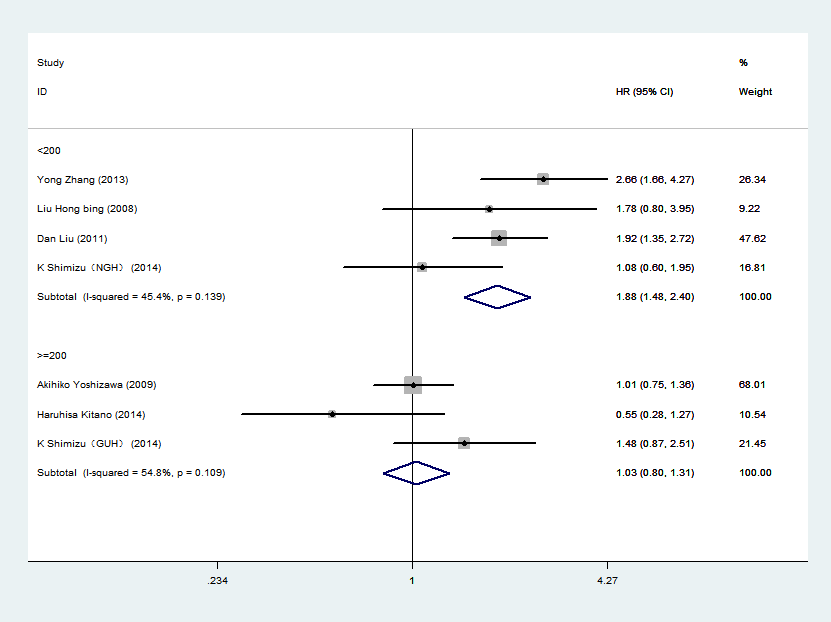

Supplement: S5 Fig — (TIF) [file pone.0116771.s007.tif]
